# The rising cost of healthcare and its contribution to the worsening disease burden in developing countries

**DOI:** 10.1016/j.amsu.2022.104683

**Published:** 2022-09-15

**Authors:** Faizan Fazal, Tayyaba Saleem, Mohammad Ebad Ur Rehman, Tehseen Haider, Abdul Rauf Khalid, Usama Tanveer, Haris Mustafa, Junaid Tanveer, Arooba Noor

**Affiliations:** Department of Medicine, Rawalpindi Medical University, Block E Satellite Town, Rawalpindi, Pakistan

**Keywords:** Healthcare, Disease burden

To the Editor,

Over the years, the disease burden of communicable and non-communicable diseases among developing countries has been on the rise. Traditionally associated with developed countries, developing countries have seen an unprecedented increase in the past years in non-communicable diseases such as diabetes, cardiovascular diseases, obesity. It was predicted that by 2020, NCD's will be responsible for about 70% of the global burden of disease, causing 7 out of 10 deaths in developing countries [1]. Infectious diseases in parallel are still largely a threat to the developing countries worldwide. Researchers have coined the term ‘Double burden of diseases’ in an attempt to explain the concurrent rise of communicable and non-communicable diseases in developing countries [2].

A review of literature shows that developing countries like Bangladesh, India, Nepal, Pakistan and Sri Lanka spend less that 4% of their GDP on healthcare [3]. This regressive budget allocation has had detrimental effects on the health of these countries. The healthcare system in these countries is underfunded, inefficient and expensive leading to a vicious cycle of increased morbidity and mortality [4].

The rising cost of healthcare is a grave problem for resource depleted countries in addition to the rising burden of diseases. It not only affects the quality of care being provided but also has led to the rationing and limiting of healthcare services [5]. Financial affordability of healthcare is one of the major barriers to healthcare seeking in resource constraint countries [6]. Out of pocket payments for healthcare services are responsible for most of the unmet medical needs in low- and middle-income countries. These payments disproportionately affect people from below the poverty line which leads to further impoverishment and adds to the disease burden [7]. Aside from the cost of healthcare itself, the indirect costs that come with obtaining healthcare such as food, lodging and transportation that discourage people [8]. Another factor that deters poor people is the prohibitive cost and access to essential life saving drugs. This also extends to vaccines for otherwise fatal diseases [9].

This situation calls for an immediate remedial and re-evaluation of our policies regarding healthcare. For developing countries lack of healthcare expenditure, allocation of budget and inadequate resource utilisation is a major problem [10]. Developing countries need to allot a higher proportion of their budget to healthcare as a positive correlation between health outcomes and health financing has been observed [11].

A greater stress on health maintenance and disease prevention is the ideal and efficient approach to counter healthcare budget and cost constraints. There also needs to be a shift of focus towards primary health care, which is a more cost-effective way of delivering health services to a wider population [12]. Globally, healthcare systems with more emphasis on primary care have shown better outcomes especially in countries with high prevalence of non-communicable diseases [13].

Funding of research programs and labs to come up with new diagnostic tests and medications in an attempt to minimize costs can also be done. Training of paramedics, nurses, and pharmacists to play a more active role in healthcare delivery can be fruitful in improving outcomes and prevent negligence [14,15].

Overall, the priority of the developing countries need to be focused around adopting a more humanised approach towards healthcare. Healthcare should be equally accessible to the poor and the rich with no discrimination in the quality of services. A bitter reality is that while the developed world progresses, developing countries health indicators worsen perpetuating the cycle of poverty and illness [16].

## Provenance and peer review

Not commissioned, externally peer reviewed.

## Sources of funding

None.

## Ethical approval

Not applicable.

## Consent

Not applicable.

## Author contribution

Faizan Fazal: Study conception, write-up, critical review and approval of the final version.

Tayyaba saleem: Study conception, write-up, critical review and approval of the final version.

Mohammad Ebad Ur Rehman: Study conception, write-up, critical review and approval of the final version.

Tehseen Haider: Study conception, critical review and approval of the final version.

Abdul rauf khalid: Study conception, write-up, critical review and approval of the final version.

Usama Tanveer: Study conception, write-up, critical review and approval of the final version.

Haris Mustafa: Study conception, write-up, critical review and approval of the final version.

Junaid Tanveer: Study conception, critical review and approval of the final version.

Arooba Noor: Study conception, write-up, critical review and approval of the final version.

## Registration of research studies

1. Name of the registry: Not applicable
2. Unique Identifying number or registration ID: Not applicable
3. Hyperlink to your specific registration (must be publicly accessible and will be checked): Not applicable

## Guarantor

Faizan Fazal.

Mohammad Ebad Ur Rehman.

## Declaration of competing interest

The authors have no conflicts of interest.
